# High-glutathione mesenchymal stem cells isolated using the FreSHtracer probe enhance cartilage regeneration in a rabbit chondral defect model

**DOI:** 10.1186/s40824-023-00398-3

**Published:** 2023-05-31

**Authors:** Gun Hee Cho, Hyun Cheol Bae, Won Young Cho, Eui Man Jeong, Hee Jung Park, Ha Ru Yang, Sun Young Wang, You Jung Kim, Dong Myung Shin, Hyung Min Chung, In Gyu Kim, Hyuk-Soo Han

**Affiliations:** 1 Department of Orthopedic Surgery, College of Medicine, Seoul National University, 101 Daehak-Ro, Jongno-Gu, Seoul, 03080 Republic of Korea; 2 Department of Orthopedic Surgery, Seoul National University Hospital, Yongondong Chongnogu, Seoul, 110-744 Republic of Korea; 3 Department of Pharmacy, College of Pharmacy, Jeju National University, Jeju Special Self-Governing Province, Jeju-do, Republic of Korea; 4 Department of Biomedical Sciences, Asan Medical Center, University of Ulsan College of Medicine, 88 Olymic-Ro 43-Gil, Songpa-Gu, Seoul, 05505 Republic of Korea; 5 Department of Stem Cell Biology, School of Medicine, Konkuk University, Seoul, 05029 Republic of Korea; 6 Laboratory for Cellular Response to Oxidative Stress, Cell2in, Inc, Seoul, 03127 Republic of Korea

**Keywords:** FreSHtracer, Glutathione, Mesenchymal stem cells, Cartilage regeneration, Chondral defect

## Abstract

**Background:**

Mesenchymal stem cells (MSCs) are a promising cell source for cartilage regeneration. However, the function of MSC can vary according to cell culture conditions, donor age, and heterogeneity of the MSC population, resulting in unregulated MSC quality control. To overcome these limitations, we previously developed a fluorescent real-time thiol tracer (FreSHtracer) that monitors cellular levels of glutathione (GSH), which are known to be closely associated with stem cell function. In this study, we investigated whether using FreSHtracer could selectively separate high-functioning MSCs based on GSH levels and evaluated the chondrogenic potential of MSCs with high GSH levels to repair cartilage defects in vivo.

**Methods:**

Flow cytometry was conducted on FreSHtracer-loaded MSCs to select cells according to their GSH levels. To determine the function of FreSHtracer-isolated MSCs, mRNA expression, migration, and CFU assays were conducted. The MSCs underwent chondrogenic differentiation, followed by analysis of chondrogenic-related gene expression. For in vivo assessment, MSCs with different cellular GSH levels or cell culture densities were injected in a rabbit chondral defect model, followed by histological analysis of cartilage-regenerated defect sites.

**Results:**

FreSHtracer successfully isolated MSCs according to GSH levels. MSCs with high cellular GSH levels showed enhanced MSC function, including stem cell marker mRNA expression, migration, CFU, and oxidant resistance. Regardless of the stem cell tissue source, FreSHtracer selectively isolated MSCs with high GSH levels and high functionality. The in vitro chondrogenic potential was the highest in pellets generated by MSCs with high GSH levels, with increased ECM formation and chondrogenic marker expression. Furthermore, the MSCs’ function was dependent on cell culture conditions, with relatively higher cell culture densities resulting in higher GSH levels. In vivo, improved cartilage repair was achieved by articular injection of MSCs with high levels of cellular GSH and MSCs cultured under high-density conditions, as confirmed by Collagen type 2 IHC, Safranin-O staining and O’Driscoll scores showing that more hyaline cartilage was formed on the defects.

**Conclusion:**

FreSHtracer selectively isolates highly functional MSCs that have enhanced in vitro chondrogenesis and in vivo hyaline cartilage regeneration, which can ultimately overcome the current limitations of MSC therapy.

**Graphical Abstract:**

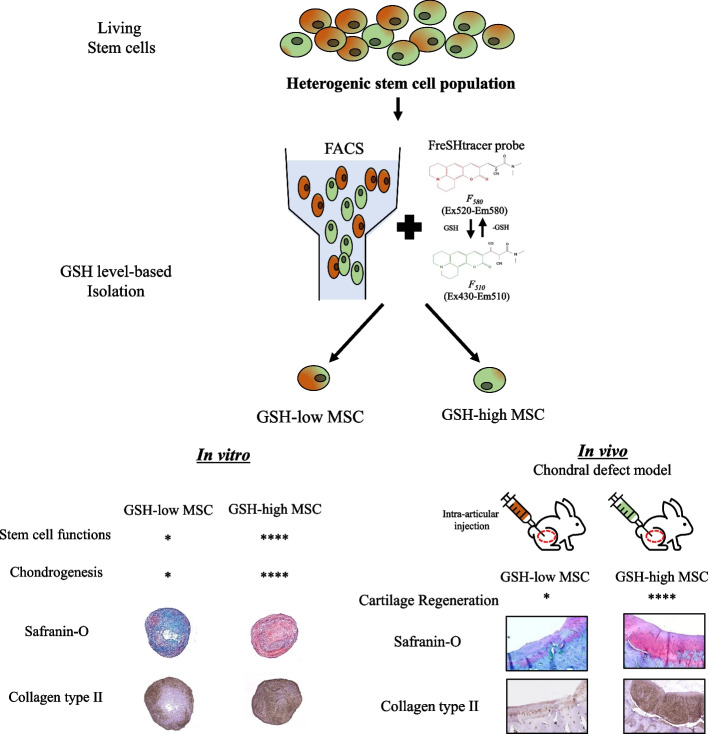

**Supplementary Information:**

The online version contains supplementary material available at 10.1186/s40824-023-00398-3.

## Introduction

The articular cartilage is an avascular connective tissue with limited self-repair capacity [1, 2]; thus, injured or damaged articular cartilage remains one of the most difficult tissues to treat. Several techniques have been developed to overcome this challenge, including bone marrow stimulation, osteochondral autografting, autologous chondrocyte implantation, and, more recently, mesenchymal stem cell (MSC) implantation [3].

Among these approaches, MSC-based therapy has been regarded as a promising therapeutic strategy for articular cartilage defects [4]. Nonetheless, certain critical limitations of MSC-based therapy remain and need to be overcome before widespread clinical application becomes possible [5]. First, there is considerable heterogeneity in living MSCs among individuals, between MSCs derived from the same tissue source, as well as between MSCs derived from the same tissue within an individual, which poses a challenge in ensuring the consistent quality of isolated stem cells for therapeutic use. Despite the potent abilities of MSCs, such challenges in quality control result in a general reluctance toward the use of MSCs in routine clinical practice. Second, MSC function is largely dependent on culture conditions [6, 7]. Indeed, our previous study confirmed that the levels of cellular glutathione (GSH) in human bone marrow-derived MSCs depended on culture conditions [8]. Furthermore, isolated MSCs undergo an age-dependent functional decline in stemness and undergo intrinsic alterations during in vitro culture, which also reduces their functions [9, 10]. This cellular alteration further raises doubts about the efficacy and reproducibility of stem cell therapy, particularly when isolating stem cells from elderly patients for regenerative purposes.

Oxidative stress results from an imbalance between the oxidative and antioxidant systems of cells and tissues, leading to the excessive production of oxidative free radicals and related reactive oxygen species (ROS) [11]. ROS are important signaling molecules that regulate cellular metabolism, proliferation, and survival [12, 13]. Reduction of oxidative stress by treating extracellular vesicles from MSCs prevented cartilage degradation and stimulated cartilage regeneration [14]. These results highlight a close relationship between oxidative stress and cellular functions.

A high cellular antioxidant GSH concentration can protect the cells from oxidative stress. GSH is the most abundant non-protein thiol that functions as an antioxidant and redox regulator in cells. GSH is present at very high concentrations (1–10 mM) in humans, allowing it to scavenge ROS either directly or indirectly [15]. GSH could be converted to its oxidized form, called glutathione disulfide, by glutathione peroxidase which decomposes ROS to H_2_O and O_2_, and the glutathione disulfide is then regenerated to GSH by glutathione reductase at the expense of NADPH [16, 17]. Therefore, measurement of the cellular GSH level has been used to evaluate the severity of oxidative stress and redox buffering capacity [12]. However, cellular GSH concentrations vary according to various conditions, which can affect the in vitro function and therapeutic efficacy of MSCs [8]. Detailed mechanistic investigations of the role of GSH in MSC function have been limited by the lack of direct and reliable tools for the real-time monitoring of dynamic changes in the GSH content of MSCs. Thus, we previously developed a reversible probe, the fluorescent real-time thiol tracer (FreSHtracer), that allows for the real-time monitoring and measurement of cellular GSH concentrations in living cells [8, 18].

**Fig. 1:**
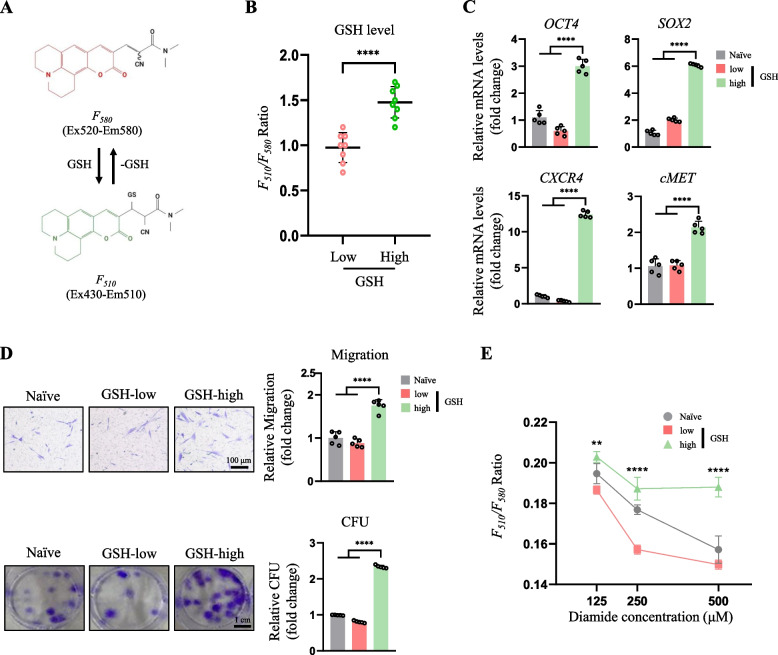
MSCs with high GSH levels isolated using FreSHtracer had enhanced stem cell function. **A** Structure of the FreSHtracer backbone and its fluorescence spectral changes upon reaction with GSH. **B** F_510_/F_580_ ratio from flow-cytometric analysis of MSCs according to cellular GSH levels (*n* = 8 each). **C** mRNA expression of stem cell markers in the hES-MSCs sorted in accordance with the levels of GSH, measured by real-time PCR (*n* = 5 per group). **D** (above) Transwell migration assay in hES-MSCs sorted based on GSH levels and in unsorted naïve cells (*n* = 5 per group). (below) Colony-forming assay in hES-MSCs sorted based on GSH levels and in unsorted naïve cells (*n* = 5 per group). **E** Evaluation of oxidant resistance of FreSHtracer-isolated hES-MSCs treated with diamide showing statistical significance when comparing GSH-high hES-MSCs to either naïve or GSH-low hES-MSCs using ANOVA. **p < 0.01, ****p < 0.0001

FreSHtracer consists of a coumarin derivative bearing a conjugated 2-cyanoacrylamide group that reacts reversibly with GSH in aqueous solutions [19], leading to a spectral shift in the maximum wavelength (λ_max_) of ultraviolet–visible absorption from 520 to 430 nm. Accordingly, in the presence of GSH, FreSHtracer will show a reduced fluorescence emission intensity at 580 nm (F_580_, λ_ex_ 520 nm) and increased fluorescence intensity at 510 nm (F_510_, λ_ex_ 430 nm; Fig. 1A) [8]. Hence, FreSHtracer enables the monitoring and comparison of cellular GSH levels based on the observed fluorescence shifts.

The positive impact of antioxidant capacity on the function of MSCs has been widely studied. Enhancing stem cell function via culturing under hypoxic condition, which is known to prime MSCs to reduce cellular ROS levels, improved chondrogenic differentiation of MSCs [20] and cartilage regeneration in both post-traumatic and focal early OA rabbit models [21]. Moreover, treatment of resveratrol, a natural antioxidant produced by plants, to MSCs significantly improved stem cell marker expression, proliferation, chondrogenic potential and in vivo cartilage regenerative potential in a rabbit osteochondral defect model [22]. Our previous study has also shown that the antioxidant capacity of MSCs plays an important role in maintaining stem cell functions, including stem cell marker expression, migration and colony forming, which can be regulated in a cellular GSH concentration-dependent manner [8]. These studies suggest that MSCs with high GSH levels would have improved chondrocyte differentiation and regenerative capacity in injured articular cartilage. Therefore, the aim of this study was to isolate MSCs based on their cellular GSH levels using FreSHtracer, and to investigate whether GSH-high MSCs have improved chondrogenic potential and regenerative capacity in vivo.

## Materials and methods

### Stem cell culture

Human embryonic stem cell-derived MSCs (hES-MSCs) were provided by Hyung-Min Chung (Konkuk University, Korea) and cultured as previously described [8]. In brief, hES-MSCs were cultured in EGM2-MV medium (Lonza, Walkersville, MD, USA) in tissue culture dishes coated with collagen (COL1A1) extracted and purified from the rat tail (Sigma-Aldrich, St. Louis, MO, USA; 150 µg per 100-pi dish).

To determine whether the effects of FreSHtracer-isolated hES-MSCs were consistent in stem cells derived from different tissues, we also examined synovium-derived MSCs (SDSCs), umbilical cord-derived MSCs (UC-MSCs), and adipose-derived MSCs (ADSCs). SDSCs were isolated from human synovial tissues from five donors, ADSCs were purchased from Sigma-Aldrich, and UC-MSCs were isolated from human umbilical cords from five donors. SDSCs and ADSCs were cultured in high glucose DMEM (Gibco, Grand Island, NY, USA) with 1% antibiotic–antimycotic solution (Gibco) and 10% fetal bovine serum. UC-MSCs were cultured in α-MEM (Gibco, Grand Island, NY, USA) with 1% antibiotic–antimycotic solution (Gibco) and 10% fetal bovine serum. The SDSCs were also divided into two groups according to the donors’ ages to determine the effect of cell aging: between 45 and 50 years (young cells) and between 70 and 80 years (old cells).

For analysis of the effect of cell culture conditions, hES-MSCs were seeded at densities of 5 × 10^3^ and 2 × 10^3^ cells/cm^2^, incubated for 19 h, stained with FreSHtracer, and the fluorescence ratio (F_510_/F_580_) was measured as described above. hES-MSCs were also cultured at densities of 2, 3, 4, and 5 × 10^3^ cells/cm^2^ followed by functional analysis for oxidant resistance at different culture conditions.

### Cellular GSH level monitoring using FreSHtracer probe

The detailed methods of synthesizing FreSHtracer probe are explained in the previous study [19]. Briefly, FreSHtracer was synthesized by mixing a coumarin-3-carbaldehyde derivative and 2-cyano-*N,N*-dimethylacetamide at 60 °C for 2 days, and purified using SiO_2_ chromatography [19]. FreSHtracer probe is now commercially available (Cell2in, Seoul, Korea). For monitoring cellular GSH levels, all MSCs were loaded with 2 µM FreSHtracer for 2 h in the culture medium and subsequently sorted at 4 °C according to their F_510_/F_580_ levels using an AriaIII Flow Cytometer System (BD Biosciences, San Jose, CA) [8]. The obtained GSH-high and GSH-low fractions were collected and prepared for further analysis. The fluorescence intensities of cells were detected at Ex405-Em525/50 and Ex561-Em582/15. The sorted cells were washed twice with 50 mL phosphate-buffered saline to remove the FreSHtracer and then further cultivated in culture medium for 24 h prior to in vitro or in vivo experiments, as described below.

### Chondrogenesis induction using pellet culture

Naïve (control), GSH-low, and GSH-high MSCs (5 × 10^5^ cells, respectively) were centrifuged at 1,500 rpm for 5 min to obtain cell pellets. The cell pellets were cultured in chondrogenic medium (low-glucose Dulbecco’s modified Eagle medium containing 0.1 mmol/L ascorbic acid 2-phosphate, 100 nmol dexamethasone, 40 g/mL proline, 100 U/mL penicillin, 100 g/mL streptomycin, and ITS Premix; BD Biosciences, Woburn, MA, USA) and supplemented with transforming growth factor beta 1 for up to 21 days. The medium was refreshed every 3–4 days. After 21 days, the pellets were harvested for subsequent analysis, including pellet morphology, size, weight, histological analysis, and mRNA expression.

### Oxidant resistance measurement

We investigated whether FreSHtracer-isolated hES-MSCs have enhanced resistance to oxidative stress by treating them with diamide, an oxidizing agent that specifically oxidizes thiol groups at indicated concentrations (125, 250 and 500 μM). The GSH levels of hES-MSCs isolated using FreSHtracer or cultured at different cell densities were monitored for 1 h following treatment with indicated amounts of diamide.

### Migration assay using transwell

Cell migration assays were performed as previously described [23]. Briefly, 8-μm-thick polycarbonate membranes were coated with 50 μL 1.0% gelatin (Sigma-Aldrich) for 1 h. MSCs were seeded at a density of 3 × 10^4^ cells/well into the upper chambers of Transwell inserts (Costar Transwell; Corning Costar, Corning, NY, USA) and the lower chamber was filled with platelet-derived growth factor-AA (R&D Systems, Minneapolis, MN, USA). After 24 h, cells that had transmigrated were fixed and stained with 0.5% crystal violet (Sigma-Aldrich). The stained cells were quantified and analyzed.

### Colony-forming unit (CFU) assay

The CFU assay was performed as previously described [23]. In brief, MSCs were re-plated at a clonal density of 60 cells/well in six-well culture plates and cultured in hES-MSC medium for 14 days. The established colonies were stained with crystal violet (Sigma-Aldrich), quantified, and analyzed.

### Quantitative real-time polymerase chain reaction (PCR) analysis

Total RNA was extracted using a TRIzol kit (Invitrogen, Carlsbad, CA, USA) and cDNA synthesis was performed using a cDNA synthesis kit (Fermentas Life Sciences) according to the manufacturer instructions. Real-time PCR was conducted for amplification with specific primers for the chondrogenic markers *COL2A1* (Forward: 5′-TTCAGCTATGGAGATGACAATC-3′ and Reverse: 5′-AGAGTCCTAGAGTGACTGAG-3′), *ACAN* (Forward: 5′-AGCCTGCGCTCCAATGACT-3′ and Reverse: 5′-TAATGGAACACGATGCCTTTCA-3′), and *SOX9* (Forward: 5′-TTCCGCGACGTGGACAT-3′ and Reverse: 5′-TCAAACTCGTTGACATCGAAGGT-3′); the hypertrophic chondrocyte marker *COLXA1* (Forward: 5′-CCCTTTTTGCTGCTAGTATCC-3′ and Reverse: 5′-CTGTTGTCCAGGTTTTCCTGGCAC-3′); and the stem cell markers *OCT4* (Forward: 5′-GAGCCCTGCACCGTCACC-3′ and Reverse: 5′-TTGATGTCCTGGGACTCCTCC-3′), *SOX2* (Forward: 5’-TACAGCATGTCCTACTCGCAGC-3’ and Reverse: 5’-GAGGAAGAGGTAACCACAGGGG-3’), *CXCR4* (Forward: 5’-ACTACACCGAGGAAATGGGCT-3’ and Reverse: 5’-CCCACAATGCCAGTTAAGAAGA-3’), and *cMET* (Forward: 5’-AGCGTCAACAGAGGGACCT-3’ and Reverse: 5’-GCAGTGAACCTCCGACTGTATG-3’) for 30 cycles using GoTaq® qPCR Master Mix (Promega, Madison, WI, USA). The gene expression level was normalized to the level of *GAPDH* (Forward: 5’-ACCCACTCCTCCACCTTTGA-3’ and Reverse 5’-TGTTGCTGTAGCCAAATTCGTT-3’) and determined using the ΔΔC_T_ method.

### Animal experiment

All procedures for animal experiments in this study were authorized by the Institutional Animal Care and Use Committee (IACUC) of Seoul National University (Approval Number: 22–0038-S1A0) and complied with the guidelines for the care and use of laboratory animals. To evaluate the in vivo therapeutic potency of the GSH-high MSCs, we generated a rabbit chondral defect model. Male New Zealand white rabbits (3.5–4.0 kg, 8 months old, *n* = 8 per group) were anesthetized by intramuscular administration of xylazine hydrochloride (5 mg/kg; Bayer) and ketamine hydrochloride (35 mg/kg; Yuhan). Anesthesia was maintained with isoflurane. The knee joints were incised using the medial parapatellar approach, and the patella was moved laterally to expose the femoral trochlear articular surface. The chondral defect (4 mm in diameter) was created in the trochlear groove of the distal femur according to a previously described method [24]. The rabbits were sacrificed 4, 8, and 12 weeks after intra-articular MSC injection to examine the regeneration of cartilage defects. The sham surgery group (referred to as normal cartilage) was sacrificed at 12 weeks, and the only-defect groups with PBS injection (referred to as only-defect) were sacrificed at 4, 8, and 12 weeks when the injection groups were sacrificed.

### Histology and immunohistochemistry

Histological analysis for extracellular matrix components that are frequently observed in chondrogenesis, such as collagen type II and glycosaminoglycans (GAGs), was carried out to evaluate the chondrogenic potential of GSH-high hES-MSCs. Rabbit knee joint tissues were fixed in 4% paraformaldehyde for 16 h, dehydrated with graded concentrations of ethanol, and embedded in paraffin. Sections (5 mm) were stained with Safranin-O/Fast Green and hematoxylin and eosin.

For immunostaining, paraffin-embedded sections were deparaffinized with xylene and dehydrated. Sections were then treated with 3% H_2_O_2_, processed with hyaluronidase, and incubated with 10% fetal bovine serum to block non-specific binding. Sections were then incubated with primary antibodies against human collagen type II and human β2 microglobulin (Abcam, Cambridge, UK) diluted in 4% bovine serum albumin for 1 h at 37 °C. Proteoglycans were detected by staining the sections with Safranin-O [25]. Images were taken at 100 × magnification and the red staining (representing the proteoglycans) in the core portion of the ligament was quantified with BIOQUANT OSTEO software, expressed as percentage of the proteoglycan (red-stained) area over the total area. Quantification of the percentage of Safranin-O positive areas was performed using ImageJ (version 1.53t, National Institutes of Health, Bethesda, MD, USA). The collagen type II staining intensity was scored using ImageJ (version 1.53t, National Institutes of Health, Bethesda, MD, USA), as follows: 0 (negative), 1 (weakly positive), 2 (moderately positive), and 3 (strongly positive). The percent of collagen type II positive area in cartilage was scored as follows: 0 (< 5%), 1 (5%–25%), 2 (25%–50%), 3 (50%–75%), and 4 (> 75%). The final score of collagen type II expression was decided by multiplying the intensity score to the positive area score (ranged 0–12).

### O’Driscoll histological assessment

Histological findings (*n* = 8 at each time point) for each section were evaluated and scored by three investigators according to O’Driscoll histological grading parameters (cell morphology, matrix staining, structural integrity, thickness/defect filling, osteochondral junction, adjacent bonding, basal integration, cellularity, clustering/distribution, and adjacent cartilage) to quantitatively evaluate the extent of cartilage repair based on the criteria of predominant tissue type, structural properties, freedom from degenerative changes in the neighboring cartilage, and freedom from cellular changes of degeneration [26]. Blinded experiments were conducted to assess histological analysis to avoid biases.

### Statistical analysis

Differences between groups of MSCs were analyzed using Student’s t-test or one-way analysis of variance. Statistical significance was set at* p* < 0.05. Results are presented as the mean ± SD. All statistical analyses were conducted using GraphPad Prism 8 software.

## Results

### FreSHtracer-isolated MSCs with high GSH levels show enhanced stem cell functions

We first investigated whether FreSHtracer could be used to monitor cellular levels of GSH in living hES-MSCs sorted by flow cytometry according to the F_510/580_ ratio. The cellular GSH concentration in the cells sorted as GSH-high hES-MSCs was significantly higher than that of cells sorted into the GSH-low group (Fig. 1B). Real-time PCR demonstrated that GSH-high hES-MSCs had significantly increased mRNA expression levels of the stem cell function-related markers *OCT4*, *SOX2*, *CXCR4,* and *cMET* compared with those of naïve and GSH-low hES-MSCs (Fig. 1C). The migration assay further showed that GSH-high hES-MSCs had increased motility compared with that of naïve and GSH-low hES-MSCs (Fig. 1D, above). The number of colonies formed by hES-MSCs was also significantly higher in the GSH-high hES-MSCs group than in the GSH-low hES-MSC group (Fig. 1D, below), demonstrating improved self-renewal ability. GSH-high hES-MSCs showed greater resistance to oxidant treatment with diamides (Fig. 1E), indicating that GSH-high hES-MSCs were more resistant to oxidative stress compared to the GSH-low group.

Since it is well known that stem cell functions decrease with donor age [27], we hypothesized that the increased proportion of the GSH-low population would be the cause of age-dependent functional decline in stem cells derived from old donors. As expected, the proportion of the GSH-low population significantly increased in SDSCs from older donors, compared with SDSCs from young donors (Supplementary Fig. 1A). In addition, stem cell marker expression was remarkably reduced in SDSCs from old donors (Supplementary Fig. 1B).

Collectively, these results indicated that the function of MSCs is dependent on cellular GSH levels, and that FreSHtracer is a powerful tool for isolating high-functioning hES-MSCs according to high GSH levels.

### MSCs with high cellular GSH levels have improved chondrogenic potential

**Fig. 2:**
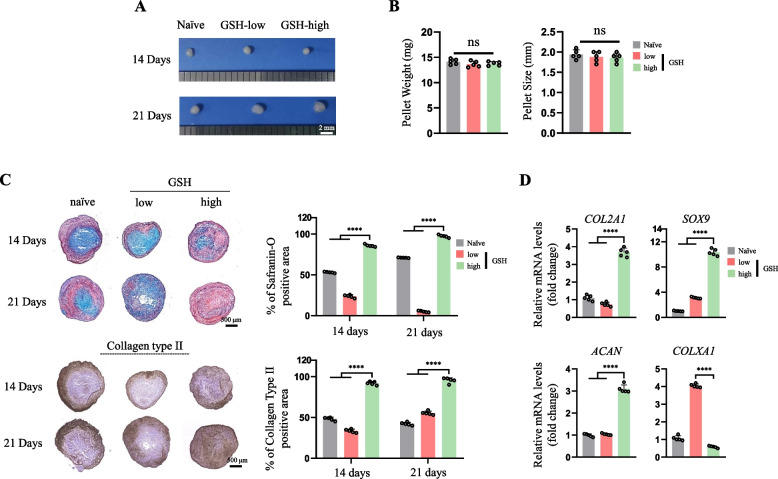
MSCs with high levels of GSH displayed enhanced chondrogenic potential. **A** Morphology of hES-MSC pellets with different GSH levels. **B** (left) Weight and (right) size of the pellets after 21 days of chondrogenic differentiation (*n* = 5 respectively). **C** Safranin-O staining for proteoglycan and immunohistochemistry staining for collagen type II after chondrogenic differentiation for 21 days using GSH-high and GSH-low hES-MSCs. **D** mRNA expression of chondrogenic markers in the hES-MSCs measured by real-time PCR (*n* = 5 per group). ****p < 0.0001

**Fig. 3:**
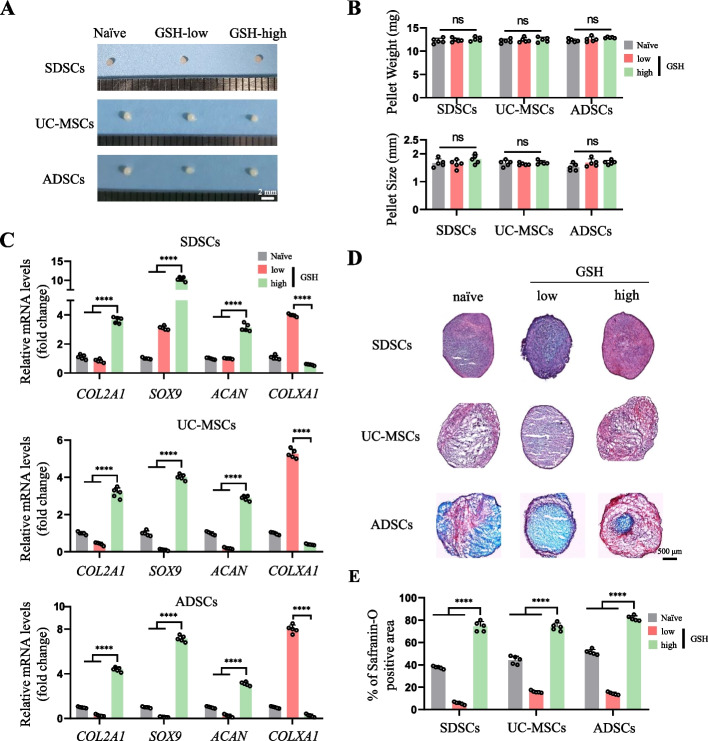
SDSCs, UC-MSCs, and BM-MSCs with high GSH levels show enhanced chondrogenesis. **A** Morphology of SDSC, UC-MSC and ADSC pellets with different GSH levels (**B**) (above) Weight and (below) size of the pellets after 21 days of chondrogenic differentiation (*n* = 5 per group, respectively). **C** mRNA levels for chondrogenic (*COL2A1, SOX9* and *ACAN*) and hypertrophic chondrocyte (*COLXA1*) markers (*n* = 5 per group). **D** Safranin-O staining of cell pellets after chondrogenic differentiation induced in SDSCs, UC-MSCs, and ADSCs. **E** Quantification of the results in (**D**) according to the positive area of Safranin-O staining (*n* = 5 each). ****p < 0.0001

Although there was no difference in size and weight among the three groups of cells (Fig. 2A and B), the pellets of GSH-high hES-MSCs harvested after 14 and 21 days of culture in chondrogenic medium showed increased production of GAGs and collagen type 2, whereas the naïve and GSH-low groups only showed light staining (Fig. 2C). Furthermore, there were significant differences in the mRNA expression levels of the chondrogenic markers *COL2A1, SOX9,* and *ACAN*, with a greater increase in GSH-high hES-MSCs compared to the other two groups (Fig. 2D). In addition, compared with that of the control (naïve) group, the mRNA level of *COLXA1*, a marker of hypertrophic chondrocytes, was significantly increased in GSH-low hES-MSCs but was decreased in GSH-high hES-MSCs (Fig. 2D). We further evaluated whether FreSHtracer isolation could be applied to other MSCs from different tissue sources (SDSCs, UC-MSCs, and ADSCs). As shown in Fig. 2A, there was no difference in size and weight between the pellets with different GSH levels in these types of MSCs (Fig. 3A and B), and chondrogenic marker expression was significantly increased in the three MSC types with high GSH levels, along with a significant decrease in *COLXA1* expression (Fig. 3C). Histological analysis with Safranin-O staining revealed that all GSH-high MSCs from different tissue sources had enhanced production of GAG, with more dense and pinkish staining compared to that of naïve and GSH-low MSCs (Fig. 3D and E). Thus, GSH-high MSCs sorted using FreSHtracer showed remarkably enhanced chondrogenic potential, regardless of the tissue of origin, indicating wide applicability of this method to various types of MSCs.

### MSCs with high GSH levels have improved hyaline cartilage regeneration in vivo

**Fig. 4:**
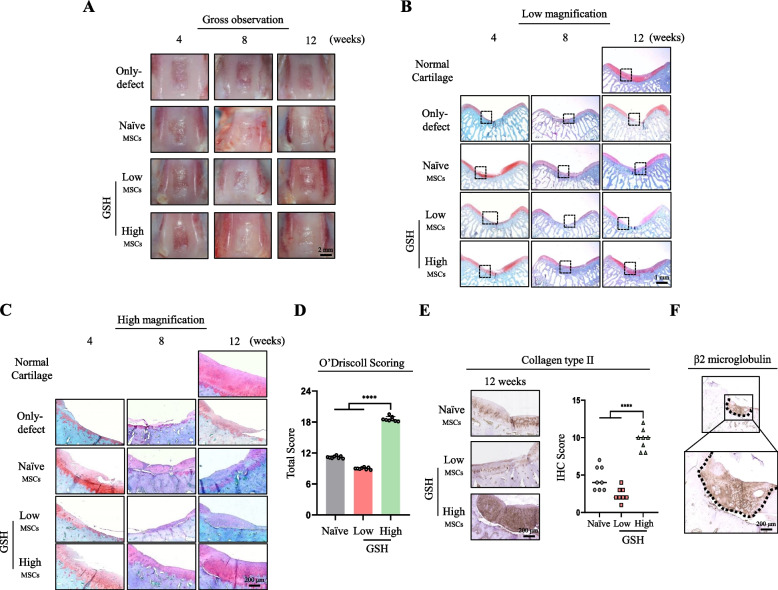
Regenerative potential of injected hES-MSCs varies based on GSH levels. **A** Macroscopic appearance of the defect lesions of the only-defect group and the treatment group which injected either naïve or GSH-low or -high hES-MSCs at 4, 8, and 12 weeks. **B** and **C** Safranin-O staining of the normal cartilage, only-defect and the treatment groups which injected either naïve or GSH-low or -high hES-MSCs at 4, 8, and 12 weeks (40 × , low magnification and 200 × , high magnification, respectively). **D** O’Driscoll scoring after injection (*n* = 8 per group). **E** (left) High-magnification image (200 × objective) to confirm collagen II expression using immunohistochemical staining and (right) quantification of collagen type II expression using scoring system (*n* = 8 per group). **F** Immunohistochemical staining of human-specific β2 microglobulin. ****p < 0.0001

To confirm that the highly functional stem cells isolated using FreSHtracer had regenerative effects on cartilage defects in vivo, we generated a 4-mm-diameter chondral defect in the trochlear groove of rabbits and compared the cartilage regenerative efficacy of GSH-high and GSH-low hES-MSCs. The only-defect group failed to regenerate during the experimental period, and the defect sites injected either naïve or GSH-low hES-MSCs were partly filled with cartilage for 12 weeks (Fig. 4A). The defect lesions injected with GSH-high hES-MSCs seemed to be recovered with cartilage during the experimental period (Fig. 4A). Histological analysis revealed that, at all time points, the naïve and GSH-low groups were filled with fibrotic cartilage or showed reduced regeneration effects (Fig. 4B and C). On the contrary, the defect sites injected with GSH-high hES-MSCs showed the greatest regenerative effects on articular cartilage, with dense Safranin-O staining, indicating that more hyaline cartilage and proteoglycans were generated on the defects (Fig. 4B and C). O’Driscoll scoring further confirmed that injection of GSH-high hES-MSCs induced hyalin cartilage regeneration in comparison to naïve and GSH-low hES-MSCs (Fig. 4D). Furthermore, COL2A1 expression was higher in the GSH-high hES-MSC-injected group, as shown by deeper staining and higher histological score (Fig. 4E).

We found strong staining of a specific antibody against human β2 microglobulin, a component of major histocompatibility complex class I molecules present on all nucleated human cells, along the lines of the lesion (Fig. 4F, dotted line). This confirmed that the injected GSH-high hES-MSCs induced a repair effect in the chondral defect sites rather than other endogenous factors, including surrounding host chondrocytes or endogenous MSCs. Collectively, these results suggested that injection of GSH-high hES-MSCs sorted by FreSHtracer lead to enhanced regenerative potential in a chondral defect animal model.

### Changes in cellular GSH levels by different cell culture densities regulate stem cell functions

**Fig. 5:**
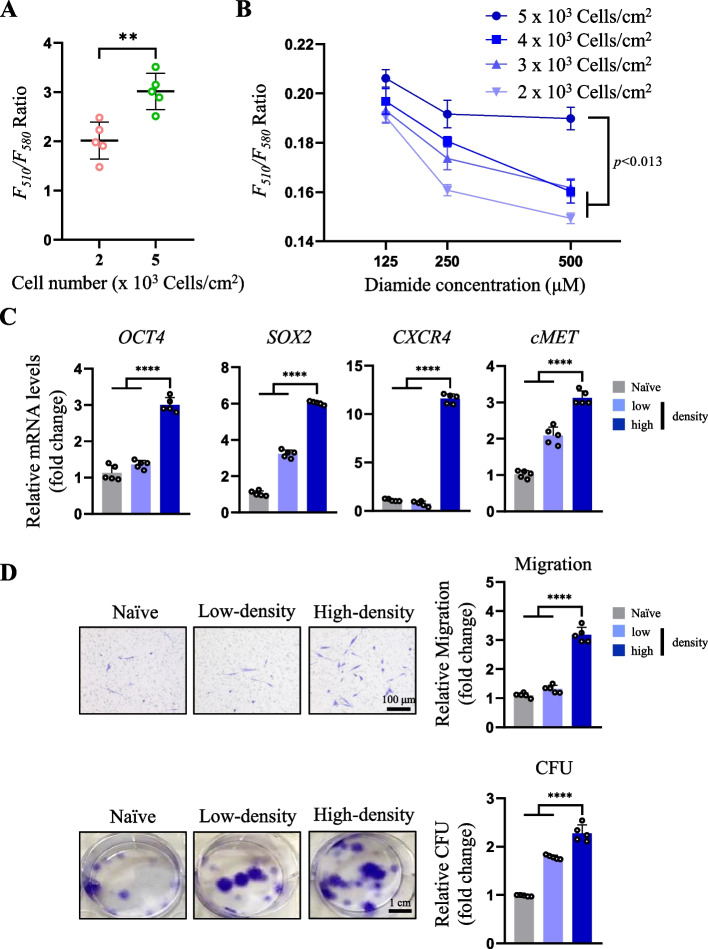
Different cell culture densities alter cellular GSH dynamics and oxidant resistance. **A** F_510_/F_580_ ratio according to cell density (*n* = 5 per group). **B** GSH level changes according to hES-MSCs culture density after diamide treatment (*n* = 5 per group). **C** mRNA expression of stem cell markers in hES-MSCs cultured at different cell densities measured by real-time PCR (*n* = 5 per group). **D** (above) Transwell migration assay in hES-MSCs cultured at different cell densities (*n* = 5 per group). (below) Colony-forming assay in hES-MSCs cultured at different cell densities (*n* = 5 per group). **p < 0.01, ****p < 0.0001

Our previous study indicated that cellular GSH levels change in a cell density-dependent manner, as GSH levels increase at relatively high cell densities [8]. Therefore, we examined the effect of cell culture density on cellular GSH levels using FreSHtracer. hES-MSCs cultured at a higher density (5 × 10^3^ cells/cm^2^) had increased GSH levels compared to those cultured at a lower density (2 × 10^3^ cells/cm^2^), confirming that GSH levels varied depending on the cell culture density (Fig. 5A). We then considered the effect of cell culture density on cellular resistance to the oxidizing reagent. The hES-MSCs cultured at 5 × 10^3^ cells/cm^2^ were the most resistant to diamide, with a higher GSH level detected with treatment of all diamide concentrations, whereas the GSH levels of cells cultured at a lower cell density dropped sharply as the diamide concentration increased (Fig. 5B). Consistently, hES-MSCs cultured at 5 × 10^3^ cells/cm^2^ (hereafter referred to as high-density) showed enhanced stem cell functions compared to those of cells cultured at 2 × 10^3^ cells/cm^2^ (hereafter referred to low-density) from all aspects, including stem cell marker expression, migration, and CFU (Fig. 5C and D). These results indicate that the appropriate stem cell culture density primed MSCs to have high levels of GSH and enhanced stem cell function.

### MSCs cultured under relatively high cell density that increases cellular GSH levels enhances hyaline cartilage regeneration

**Fig. 6:**
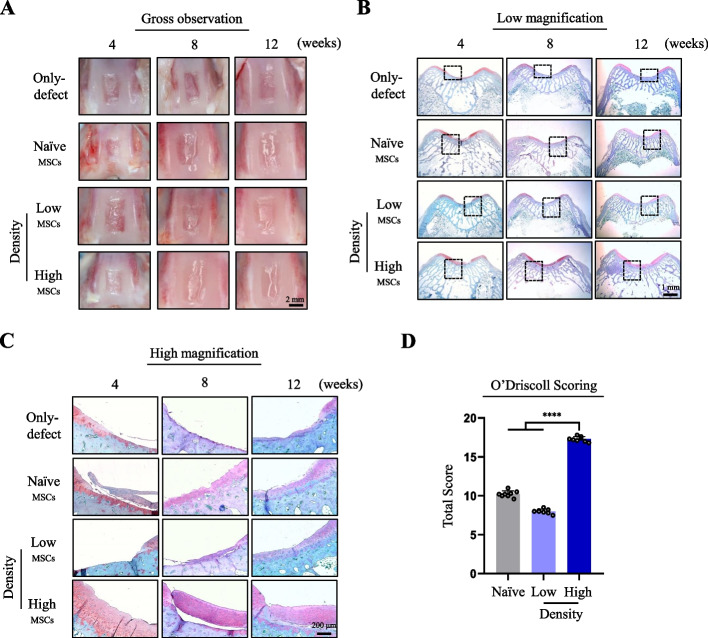
Histological changes in the chondral defect model after injection of MSCs cultured at low and high density. **A** Macroscopic appearance of the defect lesions of the only-defect group and the treatment groups which injected either naïve hES-MSCs or hES-MSCs cultured under low- or high-density conditions at 4, 8, and 12 weeks. **B** and **C** Safranin-O staining of the only-defect group and the treatment groups which injected either naïve hES-MSCs or hES-MSCs cultured under low- or high-density conditions at 4, 8, and 12 weeks (40 × , low magnification and 200 × , high magnification, respectively). **D** Scoring of cartilage regeneration parameters using the O’Driscoll scoring system after injection (*n* = 8 per group) ****p < 0.0001

To confirm that the in vivo regenerative capacity of MSCs could vary with the cell culture conditions, we cultured hES-MSCs under two different culture conditions (5 × 10^3^ cells/cm^2^; high-density and 2 × 10^3^ cells/cm^2^; low-density) and injected them into the chondral defect sites of rabbits. Similar to the results shown in Fig. 4A, gross observation revealed that the only-defect group remained damaged for 12 weeks, the naïve or low-density hES-MSC-injected groups had partly filled defect sites, and the defect sites in high-density hES-MSC-injected rabbits were filled with cartilage during the experimental period (Fig. 6A). However, at all time points, only the defect sites injected with hES-MSCs cultured at high density elicited hyaline cartilage regeneration effects, with more intense and uniform Safranin-O staining (Fig. 6B and C). O’Driscoll scoring further confirmed that cartilage regeneration was greater in the high-density group than in the low-density group (Fig. 6D). Taken together, MSCs cultured under cell culture densities that prime MSCs to possess high levels of GSH could potentiate the regeneration capacity of the articular cartilage at chondral defect sites, leading to smooth and uniform hyaline cartilage formation. Therefore, the appropriate selection of culture conditions may improve the regeneration potential of MSCs when injected in vivo.

## Discussion

ROS-mediated oxidation plays an essential role in the regulation of signaling proteins that affect the self-renewal, pluripotency, viability, and genomic stability of stem cells [28]. Cellular redox homeostasis depends on the balance between the generation and elimination of ROS by several enzymes and antioxidants, and the imbalance of cellular redox homeostasis leads to oxidative stress, which imposes a substantial burden on cells [29, 30]. Although high ROS levels cause cellular damage and dysfunction, a low basal level of ROS is considered advantageous for maintaining cellular proliferation, differentiation, and survival [31, 32]. Therefore, maintaining low levels of ROS is crucial for maintaining the self-renewal of MSCs [33], preventing mutations, and preserving their genomic and epigenomic integrity [34, 35]. GSH, the most abundant non-protein thiol in cells, acts as a redox buffer to protect cells from oxidative stress and regulate cellular redox signaling, and the thiol group of cysteine in the backbone of GSH is responsible for its antioxidant capacity in maintaining redox homeostasis [36]. In detail, cyclic adenosine monophosphate (cAMP) response element-binding protein 1 (CREB1), mediated by nuclear factor erythroid 2-related factor 2 (NRF2), was reported to be essential for the maintenance of GSH dynamics in MSCs; thereby, the CREB1-NRF2 signaling pathway potentiates the function of MSCs, which was shown to increase their therapeutic potential in a humanized mouse model of graft-versus-host disease [37]. Hence, these studies suggest that MSC functions could be regulated in an intracellular GSH-dependent manner.

Indeed, stem cell functions, including stemness and migration activities, depend on cellular GSH levels. We previously reported that MSCs with high levels of cellular GSH showed increased stem cell function and therapeutic efficiency in an asthma model [8]. The present study provides the first evidence of the functionality and regenerative potential of FreSHtracer-isolated MSCs with high GSH levels at chondral defect sites. Our in vitro and in vivo studies proved that FreSHtracer is a useful tool for the real-time monitoring of cellular GSH levels in living MSCs and for isolating MSCs based on their GSH levels. Our results showed the successful isolation of stem cells from a heterogeneous population based on cellular GSH levels without causing detrimental effects to the cells. This is achieved because of the nature of FreSHtracer, which can pass through the cell membrane and react with the thiol of GSH in the cell with high efficiency, unlike other fluorescent dyes that have several shortcomings such as irreversibility, slow kinetics [38], and low fluorescent quantum yields [39]. We found that the function of FreSHtracer-sorted MSCs was positively correlated with the levels of cellular GSH. Therefore, FreSHtracer enables the selective sorting of highly functional stem cells based on GSH levels, regardless of donor age, which may help to overcome one of the main limitations of stem cell therapy.

Numerous studies have highlighted the impact of oxidative stress on MSC differentiation into osteocytes [40] and chondrocytes [41] via the regulation of differentiation signaling cascades [42, 43]. Our results showed the increased expression of chondrogenic markers in GSH-high MSCs, including *SOX9, COL2A1*, and *ACAN*. Considering that SOX9 plays important roles in chondrogenic mesenchymal condensation in embryonic limb formation [44] and in directly controlling the expression of various chondrogenic markers such as *COL2A1* and *ACAN* [45, 46], hES-MSCs with high GSH levels also exhibited better chondrogenic differentiation potential. Although we did not assess the differentiation potential to other lineages such as osteogenesis and adipogenesis according to cellular GSH levels, we found that high GSH levels increased the chondrogenic potential of not only hES-MSCs but also of SDSCs, UC-MSCs, and ADSCs, highlighting the ability of FreSHtracer to selectively isolate highly functional stem cells regardless of the tissue source. The in vivo injection experiment in our animal chondral defect model confirmed the enhanced regenerative capacity of GSH-high hES-MSCs. Considering that stem cells maintain low levels of ROS to preserve their stemness and remain quiescent [47, 48], GSH-high hES-MSCs separated using FreSHtracer possess relatively high antioxidant activity, which reduces cellular oxidative stress, perhaps maintaining highly functional stem cells.

Although functional heterogeneity within the GSH-low hES-MSC population remains unclear, it is more likely that GSH-low hES-MSCs are more heterogeneous and may consist of a mixture of cells in various states, including senescent and unhealthy states. Alternatively, hES-MSCs with higher GSH levels could be functionally homogenous, thereby demonstrating the highly functional properties observed in this study. However, our study was conducted to compare and evaluate cell function from the bulk of FreSHtracer-sorted hES-MSCs, not at the single-cell level, which may cause doubt about the consistency of stem cell quality. Furthermore, the overlapping values of F_510_/F_580_ between the GSH-low and GSH-high populations (compare Figs. 1B and 5A) indicate a lack of standardization for sorting cells according to GSH level (high vs. low). Therefore, further studies should be conducted to obtain more information on the heterogeneity of FreSHtracer-sorted GSH-high hES-MSCs at the single-cell level and to develop assessment criteria that can more clearly distinguish stem cells with high and low GSH levels.

Our previous study revealed that cellular GSH levels differed according to cell culture density, which is known to be associated with cellular ROS production [8]. Consistently, in the present study, we found that MSCs cultured at a density of 5 × 10^3^ cells/cm^2^ exhibited the highest resistance to oxidants. Subsequent in vivo evaluation confirmed that the MSC culture conditions that induced high levels of cellular GSH elicited a remarkable regeneration capacity of hES-MSCs at the chondral defect site, suggesting that combining the selection of appropriate cell culture conditions and the FreSHtracer cell sorting method could further potentiate cartilage repair in the chondral defect model. Nonetheless, due to biological and physiological differences between a small animal model and humans, such as a thinner cartilage, possible spontaneous healing of defects, biomechanics, and physical loading in small animals, thus, an additional chondral defect repair study using minipigs should be conducted to confirm these effects.

## Conclusions

Stem cell functions rely on cellular levels of GSH, which can be monitored in real time using FreSHtracer. The functional evaluation of MSCs sorted by FreSHtracer confirmed that this probe is a suitable tool for isolating highly functional stem cells. We further confirmed the chondrogenic and regenerative capacities of MSCs isolated using FreSHtracer in vitro and in vivo. Our data indicate that cellular GSH regulates the function of MSCs, which can be sorted using FreSHtracer based on their GSH levels, and that the levels of GSH and MSC function change depending on cell culture conditions. Hence, this study suggests the wide applicability of FreSHtracer and the enhanced regenerative potential of FreSHtracer-isolated highly functional MSCs as a promising strategy to overcome the current limitations of stem cell therapy.

### Supplementary Information

**Additional file 1: Supplementary Figure 1.** Cellular GSH levels are correlated with donor age.The proportion of GSH-low and GSH-high SDSCs isolated by FreSHtracer from young and old donors.mRNA levels for stem cell markersof SDSCs obtained from young and old donors. ***p < 0.001, ****p < 0.0001.

## Data Availability

All data generated or analyzed in this study are included in this published article.
