# Correction: High-glutathione mesenchymal stem cells isolated using the FreSHtracer probe enhance cartilage regeneration in a rabbit chondral defect model

**DOI:** 10.1186/s40824-023-00452-0

**Published:** 2023-10-31

**Authors:** Gun Hee Cho, Hyun Cheol Bae, Won Young Cho, Eui Man Jeong, Hee Jung Park, Ha Ru Yang, Sun Young Wang, You Jung Kim, Dong Myung Shin, Hyung Min Chung, In Gyu Kim, Hyuk-Soo Han

**Affiliations:** 1 Department of Orthopedic Surgery, College of Medicine, Seoul National University, 101 Daehak-Ro, Jongno-Gu, Seoul, 03080 Republic of Korea; 2 Department of Orthopedic Surgery, Seoul National University Hospital, Yongondong Chongnogu, Seoul, 110-744 Republic of Korea; 3 Department of Pharmacy, College of Pharmacy, Jeju National University, Jeju Special Self-Governing Province, Jeju-do, Republic of Korea; 4 Department of Biomedical Sciences, Asan Medical Center, University of Ulsan College of Medicine, 88 Olymic-Ro 43-Gil, Songpa-Gu, Seoul, 05505 Republic of Korea; 5 Department of Stem Cell Biology, School of Medicine, Konkuk University, Seoul, 05029 Republic of Korea; 6 Laboratory for Cellular Response to Oxidative Stress, Cell2in, Inc, Seoul, 03127 Republic of Korea

**Biomaterials Research (2023) 27:54**

10.1186/s40824-023-00398-3.

The original article [1] contains an error in the Funding statement.

The corrected Funding statement can be viewed ahead in this Correction article.

