# Supplementary material for: High-glutathione mesenchymal stem cells isolated using the FreSHtracer probe enhance cartilage regeneration in a rabbit chondral defect model
Source: Biomater Res. 2023 May 31;27:54. doi: 10.1186/s40824-023-00398-3 (PMC10233867; doi:10.1186/s40824-023-00398-3)
Supplement: Supplementary file 1 — Additional file 1: Supplementary Figure 1. Cellular GSH levels are correlated with donor age.The proportion of GSH-low and GSH-high SDSCs isolated by FreSHtracer from young and old donors.mRNA levels for stem cell markersof SDSCs obtained from young and old donors. ***p < 0.001, ****p < 0.0001. [file 40824_2023_398_MOESM1_ESM.pptx]

## Slide 1
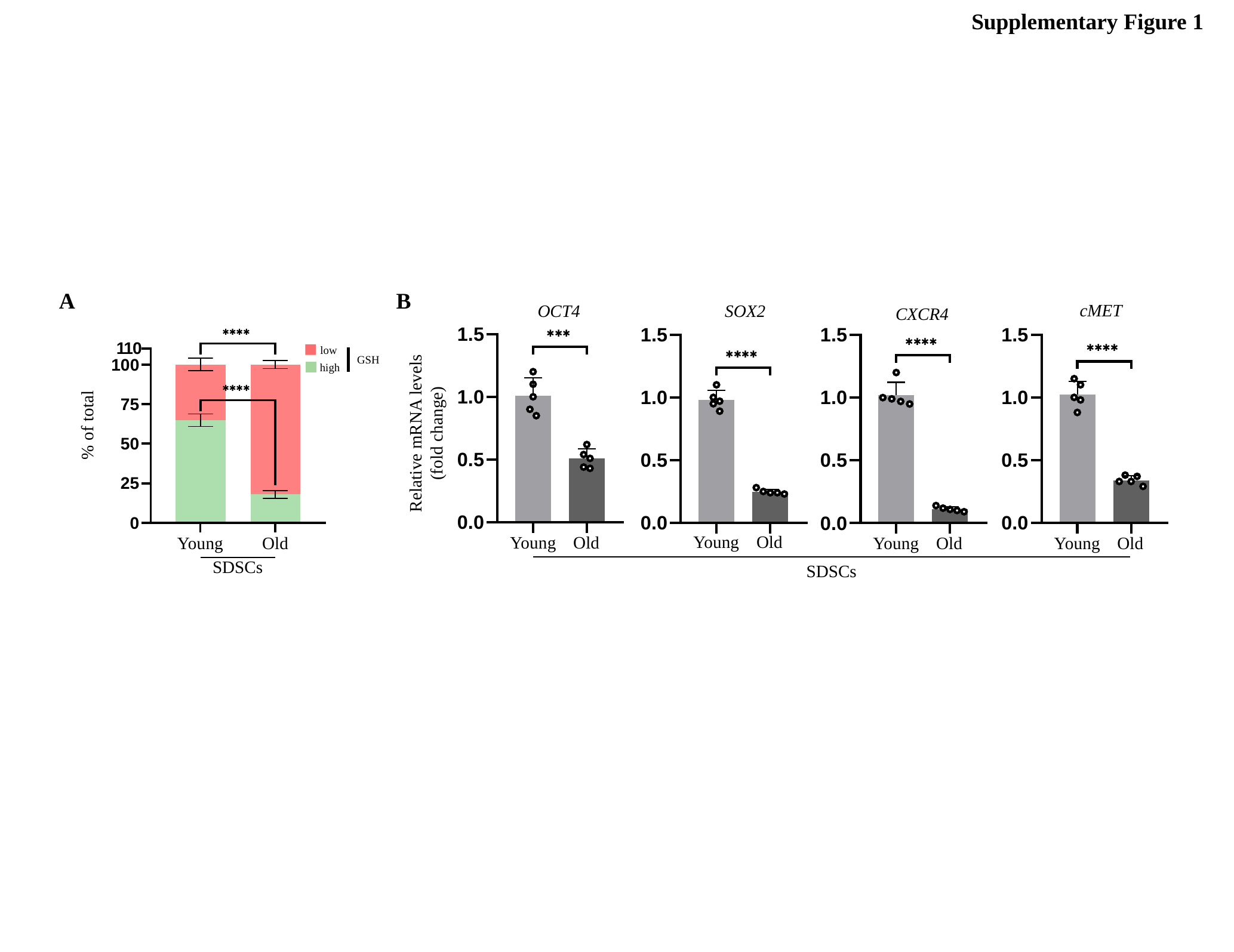

Supplementary Figure 1
A
B
cMET
SOX2
OCT4
CXCR4
Relative mRNA levels
(fold change)
Old
Young
Old
Young
Old
Young
Old
Young
SDSCs
low
GSH
high
% of total
Young
Old
SDSCs
